# Evaluation of two DNA extraction methods from maternal plasma for using in non-invasive bovine fetus gender determination

**Published:** 2012-11

**Authors:** Arash Davoudi, Alireza Tarang, Seyed Ahmad Aleyasin, Abdolreza Salehi, Ramin Seighalani, Farideh Tahmoressi

**Affiliations:** 1 *Department of Genomics and Animal, Agricultural Biotechnology Research Institute (ABRII), Branch of North Region of Iran, Rasht, Iran.*; 2 *Department of Medical Biotechnology, National Institute for Genetic Engineering and Biotechnology (NIGEB), Tehran, Iran.*; 3 *Department of Animal Sciences, Aburaihan College, Tehran University, Tehran, Iran.*

**Keywords:** *DNA extraction*, *Maternal plasma*, *Bovine fetus*, *Gender determination*, *Non-invasive*

## Abstract

**Background: **Fetal DNA in maternal plasma and serum has been shown to be a useful material for prenatal fetal sex determination during early gestational ages. Non-invasive prenatal diagnosis is now possible at 8^th^ week of pregnancy, by maternal blood sample testing.

**Objective:** The purpose of this study was to evaluate two DNA extraction methods from mother plasma and its routine clinical application in bovine fetus gender determination with non-invasive method.

**Materials and Methods:** Maternal blood samples were taken from 40 pregnant cows during the 8^th^-38^th^ weeks of gestation. DNA was extracted from 350 µl of maternal plasma with two salting-out and phenol-chloroform methods. The absorption in A_260_ and purity (A_260_/A_280_) of extracted DNA were detected by ultraviolet spectrophotometer. Three µl of the extracted DNA with phenol-chloroform method was used as a template. The PCR reaction was carried out to amplify the fragments of X and Y chromosomes of amelogenin, TSPY and BC1.2 genes.

**Results:** The difference between the mean absorption of DNA extracted by phenol-chloroform method and salting-out method was not significant in A_260_ (p>0.05, p=0.3549), but the difference between mean purity (A_260_/A_280_) of DNA extracted by phenol-chloroform method and salting-out method was significant (p<0.001). X chromosome fragment was detected in all 40 samples and Y chromosome fragments were detected in 25 plasma samples which were delivered a male calf. The sensitivity and specificity of test was 100% with no false negative and false positive results.

**Conclusion:** The results showed that phenol-chloroform method is a simple and sensitive method for isolation of fetal DNA in maternal plasma.

## Introduction

Previously, accurate prenatal diagnosis of chromosomal disorders was only available by obtaining fetal cells through invasive methods, such as chorionic villous sampling (CVS) or amniocentesis that carry a small but clear risk of miscarriage. 

Nowadays, some studies have shown that fetal DNA in maternal plasma (1) comprised a mean of 3.4% and 6.2% of total DNA in early and late gestation, respectively (2) and is clear at an extremely rapid rate following birth (3). More efficient or selective methods of plasma fetal DNA isolation should improve fetal DNA sequence detection in first trimester cases. The amount of DNA isolated from plasma is dependent on the specific method of DNA isolation employed (4, 5). 

Centrifugation speed is critical for blood separation and recovery of fetal DNA from plasma (6). Inefficient processing could lead to residual cells in the plasma and interfere with accuracy in quantification of fetal sequences (7). This likely explains the discrepancies between groups reporting on whether intact fetal cells are actually present in maternal plasma (8, 9). 

Because intact fetal cells in maternal plasma are likely apoptotic, their localization to the plasma fraction after centrifugation is likely the indirect result of these cells floating from the mononuclear cell layer due to reduced cellular density (10). Fetal sex determination is now possible at 8^th^ week of pregnancy by maternal blood sample testing and also uses for the determination of fetal gender or RhD status (11-15). High sensitivity of PCR technique allows detection of low amounts of fetal DNA in maternal plasma (15-18). This test is based on the identification of specific regions of X and Y chromosome circulating on maternal blood. 

Recent technical advances enable us to use both intact fetal cells and cell-free fetal DNA of maternal plasma and serum for non-invasive fetal gender and prenatal genetic diagnosis (18-24). Most of these techniques have improvements such as fluorescence-based polymerase chain reaction, nested PCR, multiplex PCR and real-time PCR methods, which are highly sensitive and technically demanding (14, 24-33). 

However, expensive equipments limit their application in a routine setting. Some conventional PCR analysis of maternal plasma, serum and blood using the Y-specific sequences for example; DYS14, DYZ3, and DYZ1, the Y-specific repeat sequences and sex determination region Y (SRY) have been introduced for the diagnosis of fetal gender (16-18, 23, 24, 29-34). But in a routine setting internal control of amplification for examination of results is difficult to be interpreted (30, 34). 

The purpose of this study was to evaluate two DNA extraction methods from mother plasma and its routine clinical application in gender determination with non-invasive method. 

## Materials and methods

**Blood sample preparation and maternal plasma isolation**

In this experimental study, peripheral blood samples were taken from 40 pregnant cows with gestational weeks of 8-38. Two normal cows who had no pregnant history and two normal bulls served as positive controls. 10 ml of maternal peripheral blood were collected and put into EDTA-containing tubes (20mM). Tubes were centrifuged at 1000r/min for 10 min with the brake and acceleration powers set to zero (35). 

Then tubes were re-centrifuged again at 1200r/min for 10 min with the brake and acceleration powers set to zero. Approximately 0.5ml of supernatant (i.e., the plasma) was left in the tube to ensure that the buffy coat was not disturbed. Tubes were centrifuged at 2000r/min for 5min with the brake and acceleration powers set to zero. Then 350μl of supernatant were separated and plasma samples were stored at -20^o^C until further processing.

**DNA extraction from plasma samples base on salting-out method**

350 μl maternal plasma and equal volume of purification buffer (p buffer) (NaCl 0.45 M, Tris-Hcl 1M 10mM pH=8.2, EDTA 1M 25 mM pH=8) were mixed in a 1.5ml Eppendorf tube by addition of 5μl proteinase K (20mg/ml) and 15μl SDS solutions. The mixture was placed at 56^o^C for 3h, and then 300μl of NaCl (6M) was added respectively. The mixture was stored at -20^o^C for 14h. After centrifuge (at 10000r/min for 15min), the supernatant was transferred to a fresh tube and then 2 volume of 100% ethanol was added. 

The supernatant was transferred to a fresh tube and tubes were stored at -80^o^C for 20min. Then tubes were centrifuged at 12000r/min for 10min at room temperature. The supernatant was discarded, DNA was purified and deposited with 70% ethanol, and was dried in the airing closet. Tubes were dried at 65^o^C for 3min and finally, DNA was dissolved in 20μl TE. Tubes were placed at 65^o^C for 40 minute and then stored at 4^o^C.

**DNA extraction from plasma samples base on phenol-chloroform method**

350μl maternal plasma and equal volume of TE were mixed in a 1.5 ml Eppendorf tube by addition of 5μl proteinase K (20mg/ml) solution. The mixture was placed at 56^o^C for 3 h, then 350μl of equilibrium phenol and chloroform were added respectively. The tubes were centrifuged at 12000r/min for 12 min, and then the supernatant was transferred to a fresh tube. Equal volume of chloroform and isoamyl alcohol (24:1) was added. 

After centrifuge (at 12000r/min for 12min), 1:10 of 3mol/l sodium acetate and 2 volumes of 100% ethanol was added and the mixture was stored at -20^o^C for 14h. Then tubes were centrifuged at 12000r/min for 8min at room temperature. The supernatant was discarded, DNA was purified and deposited with 70% ethanol, and was dried in the airing closet. Tubes were dried at 65^o^C for 3min and finally, DNA was dissolved in 20μl TE. Tubes were placed at 65^o^C for 40 minute and then stored at 4^o^C.

**Concentration and purity of the extracted DNA**

The concentration (absorption in A_260_) and purity (A_260_/A_280_) of extracted DNA were detected by ultraviolet spectrophotometer (NANODROP 2000 spectrophotometer, Thermo). The results were read at 260nm and 280nm.

**PCR analysis**

In this study three pair of primers (Table I) were used for amplification of X and Y chromosome sequences. Two pair primers for amelogenin gene and BC1.2 were amplified in multiplex PCR system and one pair primer for TSPY gene was amplified in conventional PCR system (36-39). Amelogenin primer was designed to amplify a 467-bp single fragment of the X-chromosome of female cattle and two 467-bp and 341-bp fragments of the X- and Y-chromosomes of male cattle. 

Also, BC1.2 and TSPY primers amplified sex-determination Y chromosome fragment (190bp and 260bp fragments, respectively) that is representative of fetal DNA. Each amplification was carried out separately (for each DNA extraction method) in a reaction volume of 25μl containing 3μl plasma DNA, 10pmol of each primers, 0.2 mM dNTPs, 1.5mM MgCl_2_ and 5U/μl *Taq* DNA polymerase (Roche), in a 0.2ml tube. 

The multiplex PCR was performed on thermo cycler (TECKNE Flexigene) with initial denaturation at 94^o^C for 5 min, followed by 35 cycles of denaturation at 94^o^C for 45 sec, annealing at 54^o^C for 60 sec and extension at 72^o^C for 60 sec. The final extension was at 72^o^C for 5min. Also, TSPY sequence was amplified in conventional PCR system by an initial denaturation step at 94^o^C for 5 min, followed by 35 cycles of denaturation at 94^o^C for 45 sec, annealing at 63^o^C for 60 sec, and extension at 72^o^C for 60sec. The final extension was at 72^o^C for 5min.

**Sequences analysis**

8μl of PCR products were mixed with 2μl loading buffer. The amplification products were analyzed by electrophoresis in 1.5% agarose gel and stained with ethidium bromide.

**Statistical analysis**

Analysis was performed by using a SAS program version 9.1. Mean comparison between purity (A_260_/A_280_) and absorption at A_260_ in both salting-out and phenol-chloroform extraction were performed by using f and *t* statistical tests. At first, equal or unequal variance was tested by *f* test. Then, mean comparisons were performed for each variable in both extraction methods by using of *t* test with unequal variances (Table II). 

## Results

**Concentration and purity of the extracted DNA by two methods**

The results of template DNA extracted from maternal blood were read at 260 nm and 280 nm respectively. The concentration and purity of DNA extracted by salting-out method were A_260_ 0.014-0.916 and A_260_/A_280_ 0.08-0.54 while these for phenol-chloroform method were A_260_ 0.034-0.261 and A_260_/A_280_ 0.93-1.03.

**Results of PCR**

**Figure 1:**
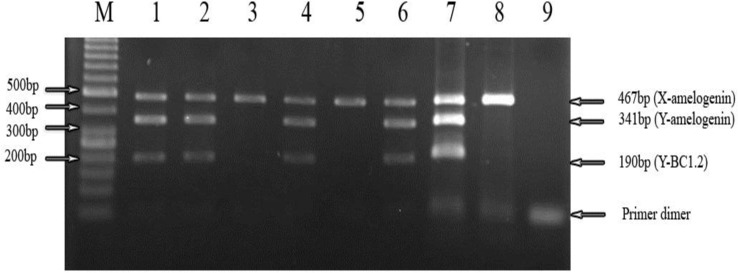
Gel electrophoresis of bovine fetal sex prediction by a simultaneous multiplex PCR analysis of maternal plasma. The multiplex amplified products of the amelogenin sequence on X chromosome, the amelogenin sequence on Y chromosome and the BC1.2 sequence on Y chromosome are 467 bp, 341 bp and 190 bp in length, respectively. Result of multiplex PCR analysis on plasma DNA samples. Lines 1-6 demonstrate the results of plasma DNA analysis of the 6 pregnant heifers. Lines 7 is an adult male, and Lines 8 is a normal heifer who had no pregnant history served as positive control. Predictions of male pregnancies were made for 1, 2, 4 and 6 and female pregnancies for 3 and 5 respectively. Line M in figure is represents the 50 base pair ladder

**Figure 2:**
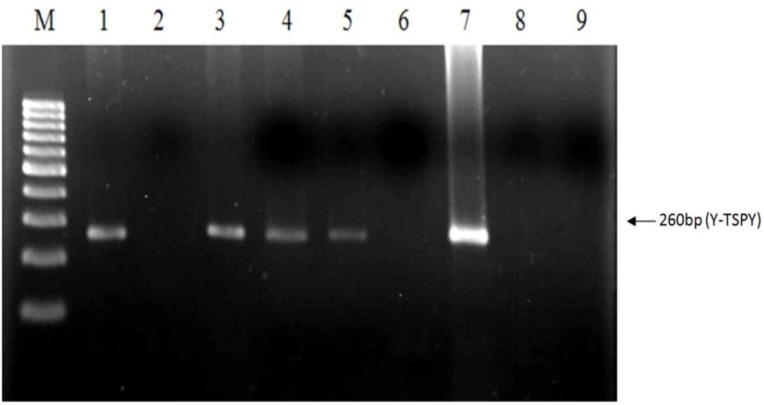
Gel electrophoresis of bovine fetal sex prediction by PCR analysis of maternal plasma. The PCR amplified products of the TSPY sequence on Y chromosome is 260 bp in length. Result of PCR analysis on plasma DNA samples: Lines 1-6 demonstrates the results of plasma DNA analysis of the 6 pregnant heifers. Lines 7 is a made with adult male, as positive control. Predictions of male pregnancies were made for 1, 3, 4, 5 and female pregnancies for 2 and 6 respectively. Line M in figs 2 represents the 100 base pair ladder.

PCR products of both DNA extraction methods were loaded on 1.5% agarose gel. No band was observed for salting-out method. The results of phenol-chloroform method were shown in figure 1 and 2. As it shown we found three clear bands at 190bp, 341bp and 467bp in the template DNA extracted from heifers bearing male fetus (Line 1, 2, 4 and 6). There was one band at 467bp after the template DNA extracted from heifers bearing female fetus, it was amplified at the same condition (Line 3 and 5). 

DNA samples extracted from normal bull and heifer that had no pregnant history respectively were amplified and, positive (Line 7 and 8) results were obtained (Figure 2). We found one clear band, 260bp in the template DNA extracted from heifers bearing male fetus (Line 2, 4, 5, 6). There was no band after the template DNA extracted from heifers bearing female fetus was amplified at the same condition (Line 3 and 7). DNA samples extracted from normal bull and heifer that had no pregnant history were amplified and, positive (Line 8 and 9) results were obtained. The results showed that fetal DNA could be found in heifers bearing male fetus.

**Result of statistical analysis**

According to Table II, The difference between the mean absorption of DNA extracted by phenol-chloroform method and salting-out method was not significant in A_260 _(p>0.05, p=0.3549), but the difference between mean purity (A_260_/A_280_) of DNA extracted by phenol-chloroform and salting-out method was significant (p<0.001).

**Table I:** Primers and their annealing temperatures for both conventional and multiplex PCR reactions

**Gene **	**Sequence**	**Forward/reverse**	**Amplified size, ** **bp**	**Annealing ** **temperature, ** ^o^ **C**
Amelogenin	5´- AAATTCTCTCACAGTCCAAG-3´ 5´- CAACAGGTAATTTTCCTTTAG-3´	Forward Reverse	467 bp 341 bp	54^o^C
BC1.2	5´-ATCAGTGCAGGGACCGAGATG-3´ 5´- AAGCAGCCGATAAACACTCCTT-3´	Forward Reverse	190 bp	54^o^C
TSPY	5´- CCCGCACCTTCCAAGTTGTG-3´ 5´- AACCTCCACCTCCTCCACGATG-3´	Forward Reverse	260 bp	63^o^C

**Table П:** Mean comparison between purity (A_260_/A_280_) and absorption at A_260_ in both salting-out and phenol-chloroform extraction were performed by using f and *t* statistical tests

**Variable **	**N**	**Mean**	**Std. deviation**	**Std. error mean**	**df**	**p-value**
A_260_	40	0.10	0.05	0.01	39	0.3549^n.s.^
A_260_/A_280_	40	0.97	0.05	0.01	39	0.001***

NS. No significant (p> 0.05)

The data in this table are expressed in Mean±SD with f and t statistical tests.

*** Significant (p<0.001)

**Table III:** Results of fetal sex prediction by non-invasive approach using the conventional PCR analysis of maternal plasma DNA in 40 pregnant heifers at various gestational ages

**Case**	**Gestational age** **(weeks)**	**Result of** **PCR**	**Birth** **outcome**	**Case**	**Gestational age** **(weeks)**	**Result of** **PCR**	**Birth** **outcome**
1	38.8	♀	F	21	12.7	♂	M
2	38.3	♂	M	22	11.9	♀	F
3	38.1	♂	M	23	11.9	♂	M
4	36.0	♀	F	24	11.6	♀	F
5	35.4	♀	F	25	11.1	♂	M
6	31.3	♂	M	26	10.8	♂	M
7	30.6	♂	M	27	10.8	♀	F
8	30.6	♂	M	28	10.2	♀	F
9	28.3	♀	F	29	10.1	♂	M
10	27.0	♂	M	30	9.9	♂	M
11	25.3	♂	M	31	9.9	♂	M
12	22.4	♂	M	32	9.3	♀	F
13	21.6	♀	F	33	9.0	♂	M
14	20.3	♂	M	34	8.9	♂	M
15	20.3	♀	F	35	8.9	♂	M
16	16.6	♂	M	36	8.9	♀	F
17	15.8	♂	M	37	8.7	♂	M
18	15.3	♂	M	38	8.2	♂	M
19	13.7	♀	F	39	8.2	♀	F
20	13.1	♂	M	40	8.1	♀	F

F= female, M= male.

## Discussion

Purity and concentration of DNA extracted by salting-out method was low, and likely because of this no band was observed in its electrophoresis. In contrast, despite the low amount of protein contamination in DNA derived from phenol-chloroform method, DNA quality was enough for PCR reaction and very clear DNA bands were observed. Although, fetus DNA which was extracted by phenol-chloroform method from maternal plasma had low amount purity (A_260_/A_280_), the gender determination was possible. Due to the nature of blood plasma, existence of some protein was expected. 

Concentration and purity of extracted DNA from plasma in this study was consistent with other studies (17). Therefor the purity and concentration of fetal DNA in maternal plasma samples was suitable for prenatal diagnosis tests. One of the benefits of DNA extraction with phenol-chloroform method is its low expense in comparison with kit method and the extracted DNA will have appropriate quality and quantity. We analyzed a total of 40 maternal plasma samples, 25 samples were carrying male fetuses, and 15 were carrying females (Table III). The Y chromosome amelogenin gene, BC1.2 and TSPY sequences were detected by PCR in 100% of pregnant cows bearing male fetuses. The sensitivity and the specificity of test were 100% with no false negative and false positive results. The results of this research showed that fetal gender is detectable through analyzing maternal plasma at early gestation. 

Though the sensitivity and the specificity of conventional PCR is less than real-time quantitative PCR method (14, 24, 32, 33), it provides a more practical approach with lower cost and acceptable sensitivity and specificity. The simultaneous amplification of amelogenin on the X chromosome and both amelogenin and BC1.2 sequences on Y chromosome in a multiplex PCR, respectively, provides a satisfactory result for bovine fetal sex determination. Multiplex PCR system was used for simultaneous amplification of 190 bp (BC1.2) and 341 bp (amelogenin) fragments which are on Y chromosome as fetal specific marker. Amplification of amelogenin gene of X chromosome was an evidence for PCR accuracy and also it used as an internal control.

Another research did not find evidence of the fetal DNA transplacental passage using btDYZ sequences in blood samples from 36 animals (gestational age 10-40 weeks) (40). The presence of a SRY gene sequence was shown in 16/20 cows with male fetuses in late pregnancy (41). Based on a previous study that detected mitochondrial DNA, another study showed the passage of fetal DNA [a Y-specific (Y-S4) and a fetal-specific (CSN2/3) sequences] to maternal circulation in mid-gestation, pre-calving, at calving and post-calving (42, 43). Another study tested the plasma of 110 pregnant cows using a SRY gene sequence. However, in the group with less than 59 days (three males and 14 females), two females were SRY positive (44). In a recent study, molecular results matched the fetal phenotypic gender in all 47 male and 37 female fetuses, including early pregnancy, and in control animals (39).

Like the results of our studies in bovine (45-46), our results demonstrated that our phenol-chloroform method and fetal gender determination using plasma is practical.

Defining of fetal gender from maternal plasma can be useful in the management of pregnant women who are heterozygous carriers of X-linked genetic disorders. This non-invasive prenatal gender determination would reduce the number of invasive procedures.

## Conclusion

The result have shown that phenol-chloroform methods is a simple and sensational method for isolation of fetal DNA in maternal blood, also, multiplex PCR is cost efficient, reliable and available for non-invasive sex determination in bovine fetus.
